# Comparative Analysis of Strength and Deformation Behavior of Cemented Tailings Backfill under Curing Temperature Effect

**DOI:** 10.3390/ma15103491

**Published:** 2022-05-12

**Authors:** Zheng Pan, Keping Zhou, Yunmin Wang, Yun Lin, Fahad Saleem

**Affiliations:** 1 School of Resources and Safety Engineering, Central South University, Changsha 410083, China; panzheng@csu.edu.cn (Z.P.); kepingzhou@csu.edu.cn (K.Z.); fahadsaleemkhan1992@gmail.com (F.S.); 2 Research Center for Mining Engineering and Technology in Cold Regions, Central South University, Changsha 410083, China; 3 State Key Laboratory of Safety and Health for Metal Mines, Maanshan 243000, China; 18336932162@163.com

**Keywords:** cement tailings backfill, temperature, unconfined compressive strength, failure characteristic, cold regions

## Abstract

Mineral resources are increasingly being developed in cold and permafrost regions. However, the mechanical and physical properties of cemented tailings backfill (CTB) cured at normal temperature are no longer applicable. To clarify the reasons for this variability, a series of tests were performed. The mechanical properties of CTB with different cement–tailings ratios (CTR, 1:4, 1:8, 1:12, 1:16, and 1:20) were tested at different curing ages (3, 7 and 28 days) and curing temperatures (20 °C, 5 °C, −5 °C, and −20 °C). The differences of CTB in mechanical and physical properties under positive- and negative-temperature curing conditions were analyzed, and the microscopic failure process of CTB under negative-temperature curing conditions was discussed. The results revealed that the mechanical properties and deformation behavior of CTB under positive- and negative-temperature curing conditions were different. The frozen CTB had higher early strength than the standard-temperature curing condition (20 °C), and the lower the temperature, the higher the early strength. The low-temperature curing condition, on the other hand, was not beneficial to CTB’s long-term strength. The low-temperature curing condition was not conducive to the long-term strength of CTB. After yielding, strain hardening and strain softening appeared in the deformation behavior of frozen backfill, indicating ductility. In contrast to the typical-temperature curing condition, the frozen CTB showed a new failure pattern that has little relation to curing time or CTR. Furthermore, the failure process of frozen backfill was reviewed and studied, which was separated into four stages, and altered as the curing time increased. The results of this study can act as a guide for filling mines in permafrost and cold climates.

## 1. Introduction

The cemented tailings filling method has been widely used in underground mining of metal and nonmetal mines around the world in recent decades [1,2,3,4]. On the one hand, this technology can handle enormous amounts of tailings waste, prevent surface collapse, maintain subterranean goaf stability, protect the safety of underground mining, and improve mining recovery rates. On the other hand, a substantial percentage of the waste is backfilled into underground goaf, which reduces the occupation of land stored on the ground by waste and the contamination of the ecological environment [5,6]. As a result, some countries have incorporated backfill mining into the industry supervision system. With the depletion of natural resources in the shallow part of the earth, the focus of resource development has shifted from the shallow to the deep part and the alpine permafrost regions in recent years, particularly in China, Russia, and Canada [7]. Because of the fragile ecological environment in the alpine regions, the cemented tailings filling technology has attracted more attention and application.

Cemented tailings backfill (CTB) is a mixture of beneficiated tailings, cementing agents, and mixing water. Usually, additives such as early strength agent, water-reducing agent, and flocculant are commonly used; they are transported to the underground goaf via pipelines. After consolidation, it can change the three-dimensional stress state of rock mass and play a certain supporting role for the surrounding rock. Therefore, the unconfined compressive strength (UCS) and deformation behavior of CTB are key indexes to evaluate its quality [8,9,10]. Many studies [11,12,13,14] have been conducted on the factors that influence these two key indicators, which can be classified into two categories: internal factors and external factors. External influences include curing temperature, curing age, stope size, and so on, while internal aspects include slurry concentration, cement–tailings ratio (CTR), binder type, additives, particle-size grading of tailings, mixing water, and their interaction. Among them, temperature is an important factor affecting the cementing process of CTB. It regulates the hydration reaction of cement, which affects physical and mechanical properties.

The high-temperature environment of deep mining urges people to pay attention to the mechanical properties of CTB under high-temperature curing conditions. Fall [15] showed that the curing temperature of 0~50 °C had a significant impact on the strength and rheological characteristics of CTB. In addition, the strength and microscopic properties of CTB are significantly affected by ultrahigh temperatures. When the temperature exceeds 400 °C, the strength of CTB decreases significantly. Wu [16] found that cement hydration is an exothermic reaction, and temperature is a critical component influencing the hydration reaction. The hydration reaction can be accelerated by raising the curing temperature. Kermani [17] investigated the UCS, porosity, and microstructure of cemented backfill containing various percentages of water glass under six curing temperatures (5~50 °C). The findings reveal that water glass can assist to improve the strength of the CTB, but the curing temperature has a greater impact on the mechanical properties of the samples. Cui [18] discovered that UCS increased linearly with the increase in curing temperature at the age of 3~7 days. At the age of 7~28 days, UCS had an exponential relationship with curing temperature, and the growth rate gradually declined. Nasir [19] incorporated temperature into the strength calculation model of CTB, which predicted well the early UCS of CTB. Xu [20] performed uniaxial compression tests on CTB samples that were cured at 20 °C, 35 °C, and 50 °C for 3, 7, and 28 days, respectively. X-ray diffraction (XRD), thermal analysis (TG/DTG), scanning electron microscopy (SEM), and mercury injection (MIP) were used to examine the microstructure and mineralogy of hardened CTB samples. The study found that at a specific curing age, the strength of CTB increases with the increase in curing temperature. Higher curing temperatures will accelerate the hydration reaction, produce more hydration products, decrease the porosity of the CTB samples, and increase the density of internal tissue. Furthermore, with the rise in curing temperature, the failure mode of CTB shifts from shear failure to tensile failure and subsequently to shear–tensile mixed failure. In terms of the rheological properties of CTB, increasing the water content and temperature of the filling slurry leads to a reduction in yield stress, which is very sensitive to temperature changes. At high temperatures, ultrafine cemented tailings backfill (UCTB) shows special rheological characteristics, which have a significant impact on pipeline transportation stability [21].

**Figure 1:**
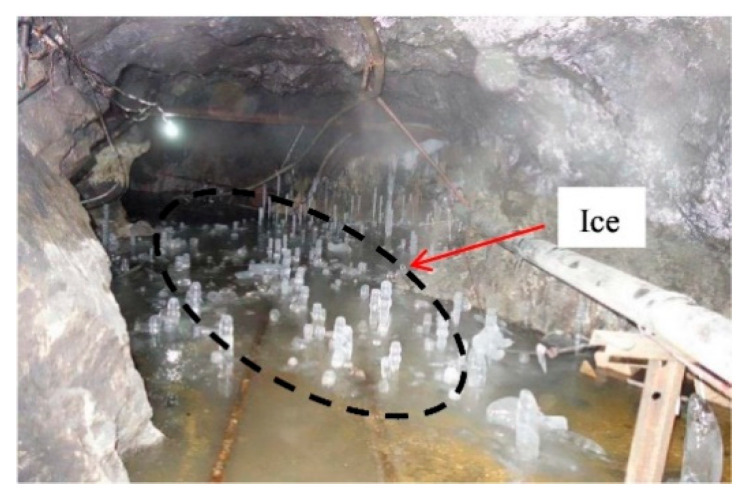
The frozen environment of underground stope [25].

It is also worth mentioning that the low-temperature frozen mining environment in the alpine regions deserves attention. For example, the Alhada lead-zinc mine in Inner Mongolia, China, has a freezing period of up to 7 months every year, where the minimum temperature can reach −38 °C. In winter, the frozen environment of underground stope can be seen by the naked eye (Figure 1). Furthermore, mining areas such as Yukon, Yellowknife, and Nunavut are also located in the cold regions. The mechanical properties of CTB are bound to be affected by the low temperature. However, the majority of studies are carried out to conserve backfill under normal- or higher-temperature curing conditions, and the conclusions may not be applicable to the application of backfill under low-temperature or even frozen conditions. Only a few studies have focused on the mechanical and physical properties of CTB at low and negative temperatures. Jiang [22] studied the rheological and mechanical properties of paste backfill under low-temperature curing conditions and discovered that the yield stress of paste backfill under low-temperature curing conditions reduced first and then subsequently increased, and was significantly lower than that under normal temperature. The strength of frozen CTB is determined by coupling influenced by curing temperature, unfrozen water content, ice content, microstructure, and other factors. The lower the curing temperature is, the higher the ice content and strength of frozen CTB. Mamert [23] investigated the influence of 3% and 5% high-early-age cement on the strength of 80% slag and 20% ordinary cement-mixed CPB in a low-temperature curing environment. The study found that while high-early-age cement was beneficial to the development of CPB strength under low-temperature curing conditions, its hydration reaction was still inhibited by low temperature. UCS is lower than in room-temperature curing conditions. Oreshkin [24] examined the physical and mechanical properties of hollow glass microsphere CTB supplemented with antifreeze at −5 °C, and the results revealed that potassium salt additive and retarder had the best effect on backfilling mortar.

Furthermore, mines in the alpine regions are often located far from cities and cement-industry-producing areas, so the price of cement itself is expensive and the transportation cost is high, significantly increasing the production cost of mining. It is estimated that the cement cost in the alpine regions can account for 75% of the cost of underground backfill. Pore ice can be employed as a special binder to improve the strength of CTB and reduce cement consumption and expense in cold regions.

To summarize, temperature is the key factor affecting the quality and cost of the backfill in the alpine regions. However, it is insufficient to evaluate the variability of CTB in positive- and negative-temperature curing conditions. In this study, the influence of different curing temperatures on the quality of CTB was evaluated, and experimental studies were carried out on CTB samples with different cement–tailings ratios (CTR, 1:4, 1:8, 1:12, 1:16, and 1:20) at different curing temperatures (20, 5, −5, and −20 °C) and different curing ages (3, 7, and 28 days).

## 2. Materials and Methods

### 2.1. Materials

#### 2.1.1. Tailings

**Figure 2:**
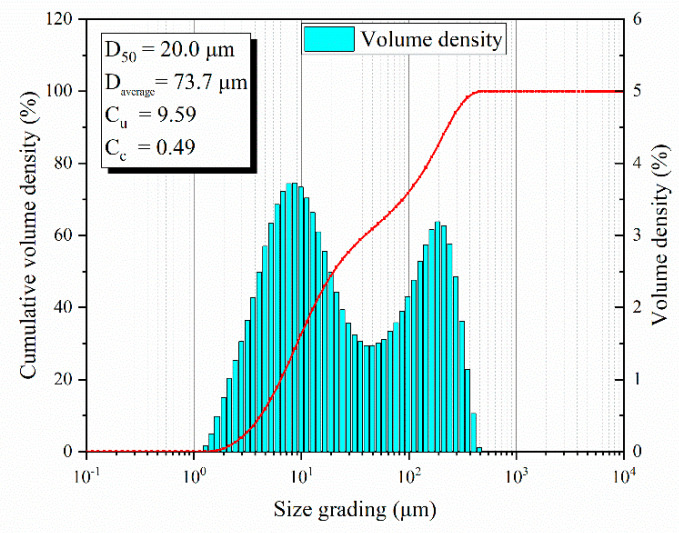
Particle-size distribution of experimental tailings.

The tailings used in this paper are provided by a mine in Inner Mongolia (a cold region), China, which adopts full tailings-cemented filling with a mass concentration of 73%. After drying and screening, the basic physical properties of the tailings were determined by a series of experiments, as shown in Table 1. The particle-size distribution of the tailings used in the test was evaluated using a Mastersizer 3000 laser particle-size analyzer, and the test results are shown in Figure 2.

#### 2.1.2. Binder

In this study, Ordinary portland cement (OPC, code P•O42.5) following the national standard of China is employed as the binding agent, which is commonly used in mines. The OPC has a bulk density of 1.184 t/m³, and the porosity is 59.92%. The chemical composition of the OPC was obtained by testing, as shown in Table 2.

#### 2.1.3. Mixing Water

Laboratory tap water (PH value about 7.4) was used to evenly mix the tailings and the binding agent, and the slurry with a mass concentration of 73% was prepared for the CTB samples.

### 2.2. Sample Preparation and Curing Conditions

**Figure 3:**
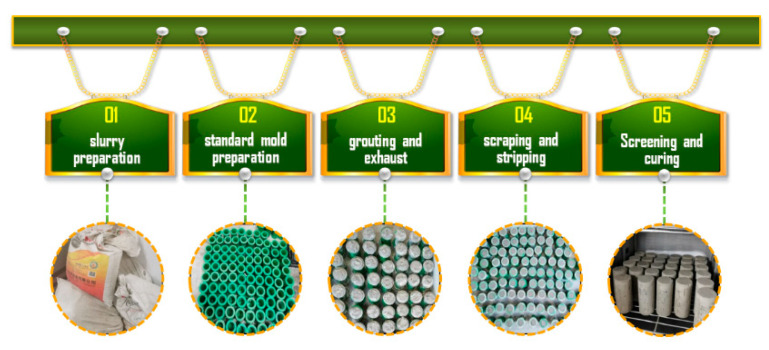
Sample preparation and curing process.

The stainless-steel container, mixer, vibrators, standard mold, and constant temperature and humidity curing box were used to prepare the CTB samples. The following are the main steps (Figure 3):

Step 1: Slurry preparation. According to the designed mass concentration, a certain proportion of tailings, binding agent, and mixing water were equally mixed in the stainless-steel container, and the mixer was used to stir clockwise and counterclockwise for 10 min.

Step 2: Standard mold preparation. The mold is a conventional cylindrical plastic mold with a diameter of 50 mm × 100 mm. First the mold was washed and dried, before a layer of lubricating oil was brushed inside the mold with a brush to facilitate demolding in the later stage.

Step 3: Grouting and exhaust. The mixture slurry was slowly poured into the mold and placed on the vibration table to discharge bubbles. The filling slurry was then continued to be added to the mold until the slurry was partly higher than the mold, to prevent settlement.

Step 4: Scraping and stripping. The slurry was scraped flat with a scraper after it had initially been set, then left to stand for 24 h. Then the CTB samples had a certain strength and could remain independent, and the samples were demolded.

Step 5: Screening and curing. After samples’ demolding, the diameter and height of the samples were measured with vernier calipers, and the samples with large deviations were removed. According to the test plan, the same conditions were prepared for 3 samples. Qualified samples were numbered and grouped, and placed in a constant temperature and humidity curing box (Table 3).

### 2.3. Testing Method

#### 2.3.1. Unconfined Compressive Strength (UCS) Test

It is critical to precisely measure the UCS of the CTB samples as a key index for evaluating the quality of CTB. The UCS with the specified ages is measured using the WHY-300/10 microcomputer-controlled automatic pressure testing machine. The maximum load is 300 KN and the loading speed is 1.2 mm/min. It should be noted that it is important to ensure that the loading temperature of CTB is the same as the curing temperature. 

#### 2.3.2. Physical and Microstructure Tests

Tescan VEGA3 LMU tungsten filament scanning electron microscope (SEM) was used to observe the microstructure and macroscopic phase of the CTB. Advance D8 X-ray diffractometer (XRD) was used to analyze the physical diffraction pattern of the raw material and hydration products, analyze the composition of hydration products, and compare the gap before and after the reaction. Before conducting SEM and XRD experiments, some samples were chosen from the damaged samples, and the hydration reaction was terminated with anhydrous ethanol and dried at 45 °C for 24 h.

## 3. Results and Analysis

### 3.1. Effects of Temperature on UCS Development in CTB

**Figure 4:**
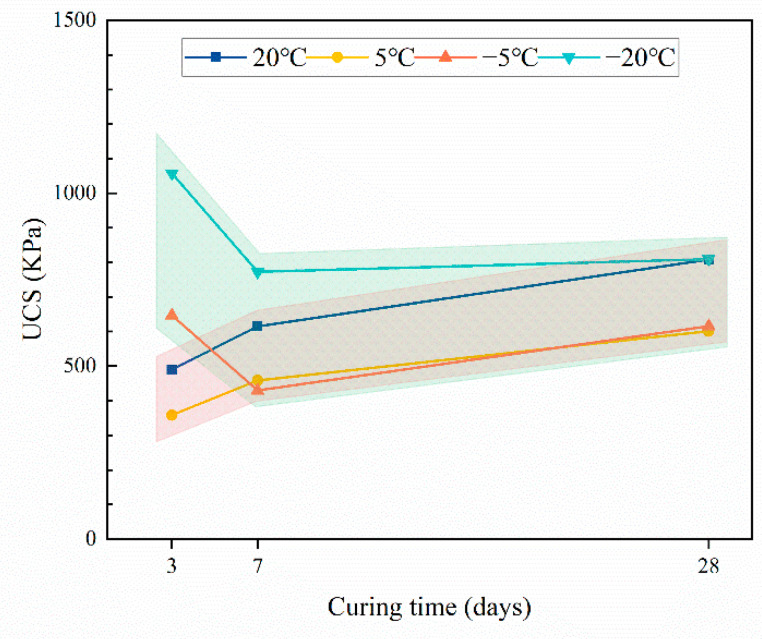
Evolution characteristics of CTB strength with curing time (CTR is 1:12).

The experimental results revealed that the temperature has a significant impact on the strength development of CTB. Regardless of the CTR, the variation pattern of CTB strength development with curing temperature and curing time is consistent. Temperature plays a vital role in CTB strength development, especially early strength. Figure 4 depicts the effect of the curing temperature on the strength of CTB at different curing ages (CTR is 1:12, for example). It can be seen that there is a significant difference in the effect of positive- and negative-temperature curing conditions on the development of strength with the curing time.

When the curing temperature is positive, the strength of CTB increases gradually with the increase in curing time, and the strength growth rate is faster before 7 days and slows down after 7 days. This is closely linked to the hydration reaction rate of cement. After the preparation of CTB, dicalcium silicate (C_2_S) and tricalcium silicate (C_3_S) in the cement clinker is dissolved quickly to generate ettringite (AFt), calcium hydroxide (CH). and calcium silicate hydrate (C-S-H) gel with cementation. The hydration products bind the tailings particles together, while CTB provides strength. The cement-clinker level in CTB is high during the curing process from 3 to 7 days, the hydration response rate is fast, hydration products build quickly, and CTB strength increases rapidly. With the increase in curing time, the cement clinker without hydration reaction of CTB decreases, the hydration reaction rate drops, and the strength growth of CTB slows down [26]. The UCS of CTB is 490.15 Kpa, 614.82 Kpa, and 808.41 KPa at 3 d, 7 d, and 28 d under the curing condition of 20 °C, respectively, and when the curing temperature is reduced to 5 °C, the strength of CTB is 358.06 KPa, 458.76 Kpa, and 601.07 Kpa, respectively, decreasing by 26.95%, 25.38%, and 25.65%. This indicates that the decrease in curing temperature is detrimental to the cement-hydration reaction and low temperatures have an inhibitory effect on the cement-hydration reaction [27]. However, it does not seem to affect the hydration reaction rate of cement, and its strength growth pattern is essentially consistent with that of curing at 20 °C.

It is noteworthy that the strength development trend of CTB changes when the curing temperature drops below 0 °C. The curing temperature below 0 °C conferred the early strength of CTB, and the lower the curing temperature, the higher the early strength of CTB. Compared to the normal-temperature curing conditions at 3 days, the strength of CTB increased by 31.98% and 115.77% at −5 °C and −20 °C, respectively, which is a considerable increase in strength. This is because the pore-water phase of the filling body freezes into solid ice, and ice formation acts as a kind of cementation that leads to the increase in the early strength of CTB. In contrast to positive-temperature curing, the strength of CTB decreases first and then increases under negative-temperature curing. In reality, while low-temperature freezing conditions provided a high early strength, they were not conducive to the long-term strength growth of CTB, and the late strength growth was slow due to a considerable reduction in the hydration reaction rate. It can be seen that the frozen-environment conditions have two mutually exclusive effects on the strength development of CTB. On the one hand, liquid water is converted to solid ice, and ice’s cementing ability causes CTB to gain strength quickly; on the other hand, the low-temperature freezing environment inhibits the hydration reaction, and the generation of ice further reduces the content of liquid water involved in the hydration reaction, which is detrimental to the long-term strength of CTB.

**Figure 5:**
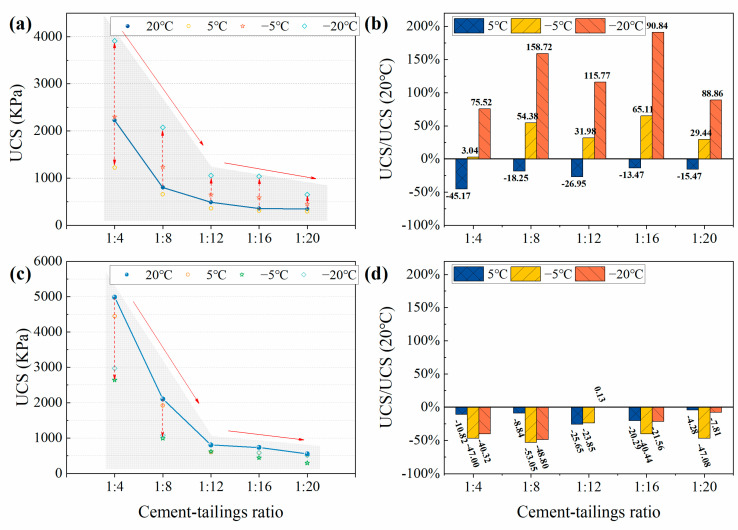
The relationship between CTR and the UCS of CTB at different curing temperatures and the strength change compared with curing at 20 °C, (**a**,**b**) 3 days, (**c**,**d**) 28 days.

As it is known, the UCS of CTB is closely related to CTR, and Figure 5 reflects the strength development trend of CTB under different curing temperatures as well as the strength change compared with the normal-temperature curing conditions. The experimental results show that regardless of the curing temperatures, the UCS of CTB decreases gradually as the CTR decreases, and the UCS of CTB does not decrease significantly when the CTR is less than 1:12, indicating that the CTR is not the main reason affecting the UCS, as found in relevant studies [28,29]. It should be noted that temperature has a substantial effect on the early and long-term strength of CTB with varied CTR.

At 3 days, the UCS of CTB reduced at 5 °C versus 20 °C curing conditions. When the CTR was 1:4, 1:8, 1:12, 1:16, and 1:20, the UCS decreased by 45.17%, 18.25%, 26.95%, 13.47%, and 15.14%, respectively. Under negative-temperature curing conditions, the UCS of CTB showed significant anomalous enhancement, with the enhancement range extending from 0% to 200%. The lower the curing temperature, the greater the strength growth. Under the condition of −20 °C, the UCS of CTB with a CTR of 1:16 increases by 190.84%, which is beneficial to the rapid strength formation of the backfill in the underground goaf in cold regions. It is determined that the negative-temperature curing condition is favorable to the rapid freezing and solidification of pore water, and the liquid pore-water phase transforms into solid pore ice, and the ice cements the tailings particles surrounding it, which contributes a positive role in the early strength of CTB. The test results demonstrate that the cementing ability of pore ice is much higher than that of the cement-hydration reaction at 3 days.

At 28 days, the UCS of CTB at low-temperature curing conditions was reduced to varying degrees as compared to 20 °C. There was no significant correlation shown between the curing temperature and CTR. The UCS reduction in CTB was most obvious at −5 °C. The UCS was reduced by 47.00%, 53.05%, 23.85%, 40.44%, and 47.08% for CTR of 1:4, 1:8, 1:12, 1:16, and 1:20, respectively. This indicates that the low-temperature curing conditions are not conducive to the long-term strength of CTB. The analysis concludes that the low temperature and frozen curing environment inhibited the hydration reaction, but did not stop the hydration reaction. At 28 days, the cementation capacity of the hydration reaction products exceeded that of the pore ice, and the lower the curing temperature, the stronger the inhibition of the cement-hydration reaction. Under freezing conditions, the pore ice and hydration products mutually contribute to the UCS of CTB, and the ice content at −20 °C is greater than that at −5 °C. Therefore, the UCS of CTB is reduced the most at −5 °C.

### 3.2. Effects of Temperature on the Deformation Behavior in CTB

**Figure 6:**
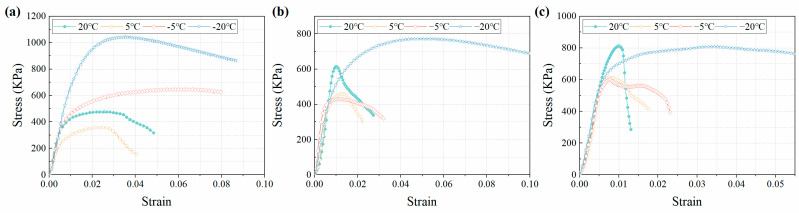
Stress–strain characteristics of CTB with CTR of 1:12 at different curing temperatures. (**a**) 3 days, (**b**) 7 days, (**c**) 28 days.

In addition to UCS, the deformation behavior of CTB is an important key factor affecting the stability of backfilled underground goaf. To illustrate the effect of temperature on the deformation behavior of CTB, typical results of the stress–strain behavior of CTB specimens with CTR of 1:12 at different curing ages are illustrated in this study (Figure 6). It can be seen that the effect of temperature on the deformation behavior of CTB is obvious, especially in the early curing time. At the same strain rate, CTB develops more plasticity and ductility with the decrease in the curing temperature, and strain-hardening and strain-softening phenomena occur simultaneously. The deformation behavior of CTB differs between positive- and negative-temperature curing conditions and is directly related to the curing ages.

When the curing temperature is above 0 °C, the UCS of CTB arises from the cementation of the cement-hydration products on the tailing aggregates. When the curing temperature decreases, the rate of hydration reaction is inhibited and the production of hydration products is slow; therefore, the UCS cured at 20 °C is higher than that at 5 °C, whether at 3, 7, or 28 days. At 3 days, the UCS is very soft due to the low hydration and exhibits more plastic behavior (Figure 6a). This similar behavior was discovered in the cemented paste backfill [30]. After reaching the yield point, CTB maintains a high strength for a certain period before failing. At this stage, CTB has fewer hydration products and weak cementation ability. Under the action of axial pressure, the cemented particle body first loses the bearing capacity and CTB exhibits yielding. To maintain the stability of CTB, the pressure drives the particles within it to agglomerate through friction. When the pressure increases further, the cracks inside CTB initiate and develop until CTB failure. With the growth of the curing time, the water in the CTB matrix is gradually consumed and the precipitated hydration products increase, shortening the post-peak deformation time of CTB, increasing the peak stress, and the stress decays rapidly after reaching the peak (Figure 6c).

The stress–strain curve features of CTB are obviously different when the curing temperature is below 0 °C compared to positive-temperature curing conditions. There is no obvious elastic stage in the stress–strain curve of the frozen CTB. At the initial stage of strain, the stress growth rate is relatively high, and with the increase in strain, the stress growth rate decreases and tends to zero. The stress–strain curve exhibits obvious strain-hardening and strain-softening characteristics. At 3 days, CTB cured at −5 °C exhibited a viscoelastic-plastic type of stress–strain relationship with no apparent elastic yield point and a significant strain-hardening behavior, while at 7 and 28 days they exhibited strain-softening behavior. The CTB cured at −20 °C all exhibited strain-softening behavior and had high peak stress and strain characteristics, independent of the curing time. Furthermore, after the stress reaches the peak, CTB retains a high strength for a long time under continuous loading. The presence of ice and cohesive unfrozen water in frozen soils has been researched, and this deformation characteristic is assumed to be connected to the coexistence of ice and cohesive unfrozen water in frozen soils. In comparison with frozen soil, the strength of the frozen CTB is additionally related to the cementation force provided by hydration products, the internal friction between particles, and the coupling of hydration products with pore ice. Therefore, when the pore ice loses strength, the cementation force and internal friction between the hydration products and the particles compensate for this resistance loss. This mechanism also explains the strain-hardening behavior of CTB under negative-temperature curing conditions. However, the ice becomes stronger when the curing temperature is as low as −20 °C, resulting in higher peak stress. Moreover, the cementation force between the pore ice and the tailings particles is reduced by melting and breaking under the effect of pressure [25]. It explains why the CTB exhibits significant strain-softening behavior at 3, 7, and 28 days when cured at −20 °C.

### 3.3. Effect of Temperature on Failure Characteristics of CTB

**Figure 7:**
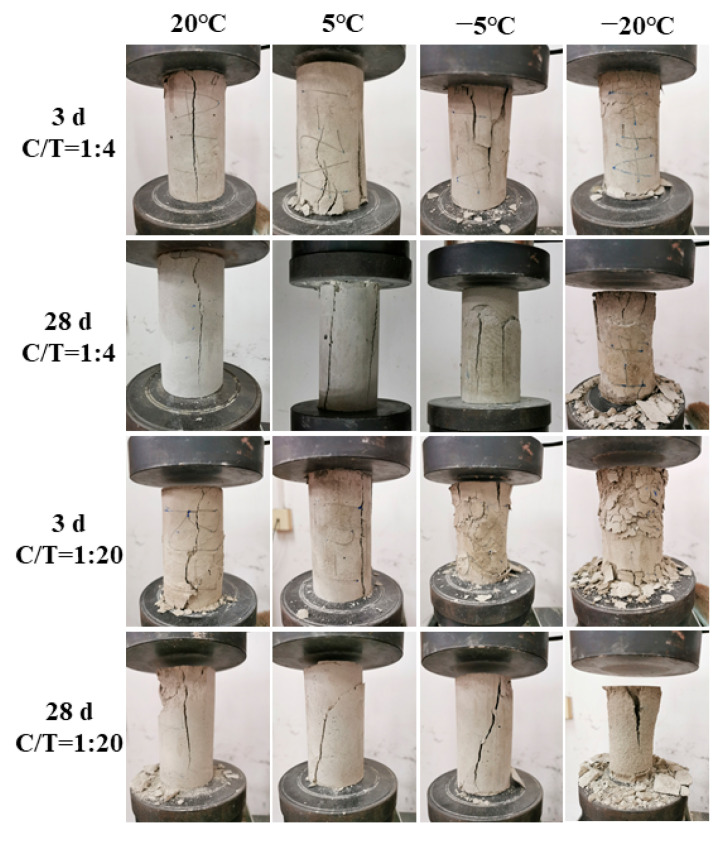
Failure photographs of CTB specimens at different curing times. (CTR of 1:4 and 1:20, curing temperatures are 20, 5, −5, and −20 °C).

The failure characteristics of CTB must be clarified to guide the filling of underground extraction areas. The previous analysis indicates that temperature has a significant effect on the strength development and deformation characteristics of CTB. The mechanical behavior of CTB differs significantly under positive- and negative-temperature curing conditions, especially in the early curing time. Therefore, CTB specimens with CTR of 1:4 and 1:20 were chosen to observe their early and long-term failure characteristics under different curing temperatures (Figure 7).

It can be seen that under the action of axial pressure, the CTB exhibits a variety of failure characteristics, such as single-crack splitting failure, multiple-cracks splitting failure, single-inclined-plane shear failure, and double-inclined-plane shear failure, which is consistent with most research conclusions on the failure characteristics of CTB [31,32]. The difference in this study is that a new failure characteristic, plastic tensile failure, appears in this experiment. The surface of the CTB specimen is fractured and falls off, and considerable axial and radial deformation occurs at both ends of the specimen, indicating strong compressibility overall. Several minor cracks are extending from the end along the axial direction, but there is no penetration, and the sample integrity and binding are satisfactory. This feature is similar to the waist-drum failure of frozen soil [33,34,35]. It should be mentioned that the failure behavior of CTB epidermis rupture and peeling is caused by the stress difference between the surface and the interior under the load. During the UCS test, heat exchange occurs between the CTB specimen and the environment, resulting in a temperature difference between the surface peel and the interior of the CTB.

Under positive-temperature curing conditions, CTB mainly exhibits single-crack splitting failure, multiple-cracks splitting failure and single-inclined-plane shear failure, regardless of CTR and curing age. While under the negative-temperature curing condition, CTB mainly exhibits single-inclined-plane shear failure, double-inclined-plane shear failure, and plastic tensile failure. The higher the CTR and the longer the curing age, the more likely CTB exhibits splitting failure. The lower the CTR, the more it exhibits shear failure. This is because higher cement concentration results in more hydration products, which form a harder cementitious body in the CTB. Under the action of sustained load, the tensile stress generated by the Poisson effect in the CTB firstly reaches the tension limit of the soft structural plane. When the CTR decreases, the CTB stress first reaches the shear stress limit of the weak structural plane.

With the decrease in the curing temperature, the failure characteristics of CTB evolve from splitting failure to shear failure and then to plastic tensile failure. Under the negative-temperature curing condition, when the CTR is higher or the curing time is longer, the CTB mainly shows shear failure. However, when the curing temperature drops to −20 °C, the failure characteristics exhibit plastic tensile failure, which appears to be unrelated to CTR and curing age. This phenomenon occurs because of the coupling effect of the pore ice and the hydration reaction, which gives CTB a high resistance to load deformation. Furthermore, when subjected to higher stresses, the internal particles of CTB are prone to axial and radial slip, resulting in larger plastic deformation due to the coexistence of pore ice and unfrozen water. Fractures are formed when stress surpasses the cementation force between pore ice and tailings, which is macroscopically manifested by the initiation and prolongation of fine cracks at the end of the CTB.

## 4. Discussion

Temperature is thought to have a catalytic effect on the hydration reaction rate. The hydration reaction is impeded at lower curing temperatures, resulting in decreased CTB strength. However, the production of ice in CTB breaks the link between conventional mechanical characteristics and physical properties when the curing temperature is sub-zero. The participation of the pore ice complicates the strength development and deformation behavior of CTB

**Figure 8:**
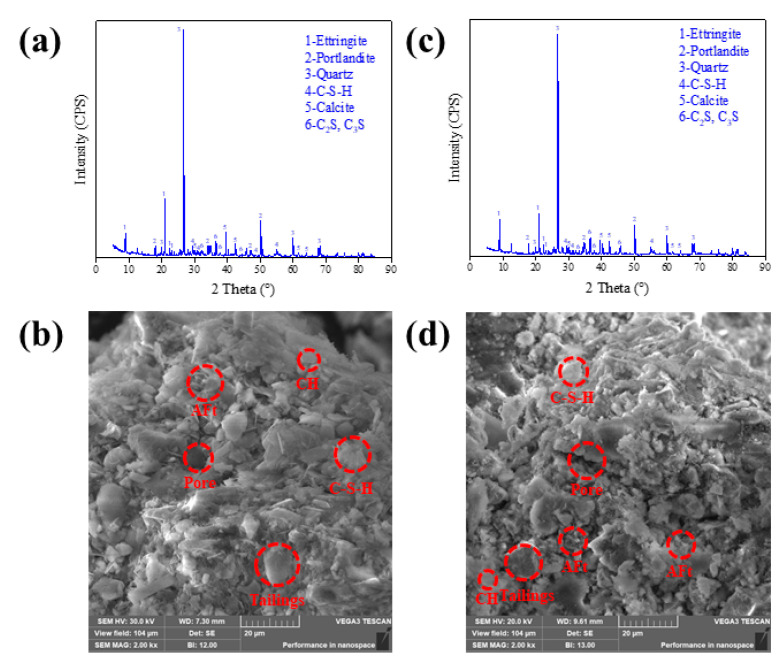
XRD patterns and SEM images of CTB with CTR of 1:12 at curing time of 3 days :(**a**,**b**) 20 °C, (**c**,**d**) −20 °C.

The experimental data have confirmed that temperature has a significant effect on the early-age characteristics of CTB, so the specimens at the CTR of 1:12 curing for 3 days are selected for further discussion. The research has shown that the strength development of CTB is dependent on the generation and content of the hydration products such as C-S-H gel, AFt, CH, etc. [36]. The XRD pattern and SEM images of CTB are shown in Figure 8 to further analyze the temperature effect on the strength development of CTB.

Analysis shows that the main phases in CTB can be divided into two types of substances after curing for 3 d: one is the intrinsic phase, such as quartz, dicalcium silicate (C_2_S), tricalcium silicate (C_3_S), etc.; the other is the secondary phase, such as ettringite (AFt), calcium hydroxide (CH), hydrated calcium silicate (C-S-H), calcium carbonate, etc. The diffraction peaks of C_2_S and C_3_S indicate that the hydration reaction is not complete and there is cement clinker that has not been hydrated. The XRD diffraction patterns showed the characteristic peaks of AFt, C-S-H, and CH under low-temperature curing conditions, which are similar to the characteristic peaks under normal-temperature curing conditions, but with the peak intensity reduced, indicating that low-temperature curing conditions can only slow down the rate of the hydration reaction but not prevent it. Combined with the SEM images, it is clear that under the negative-temperature curing conditions, the amount of AFt and C-S-H production that contributes to the early strength of CTB is reduced. Furthermore, it can be observed that the percentage of pores in the SEM images under negative-temperature curing conditions is relatively larger, indicating the space occupied by the pore-ice melting. It is fully demonstrated that the development of CTB strength under negative-temperature conditions is the result of the coupling effect of cement-hydration reaction and pore ice, but it is not able to quantitatively characterize the cementing ability of ice and cement-hydration products.

The stress–strain curves show that there is a significant difference between the deformation characteristics of CTB under negative- and positive-temperature curing conditions. The presence of ice makes the deformation characteristics of CTB under negative-temperature curing conditions more complicated. In a negative-temperature environment, liquid water quickly solidifies into solid ice, forming ice cement in CTB. The cohesion of ice cement is proportional to the ice crystal content, which depends on the curing temperature. Therefore, the deformation characteristics of CTB have a high temperature dependence. With the decrease in curing temperature, the deformation characteristics of CTB vary from elastic-plastic to viscoelastic-plastic to strain-hardening to strain-softening.

**Figure 9:**
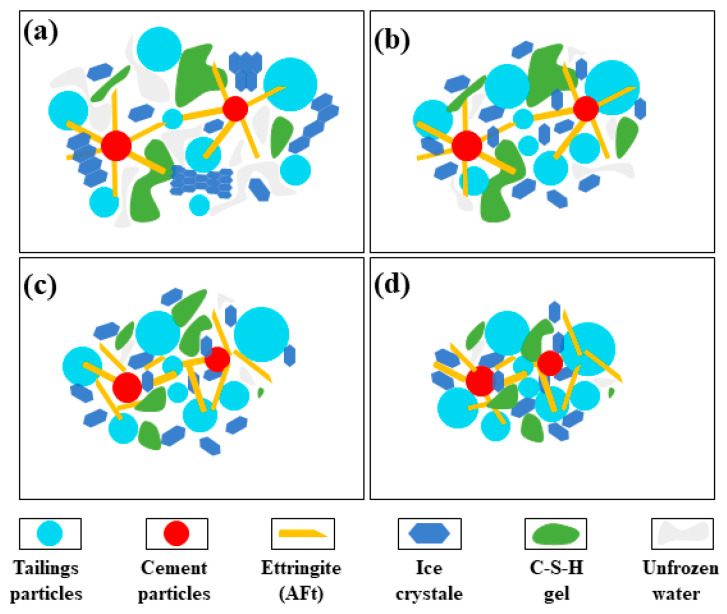
Failure process of frozen CTB (the frozen CTB is a mixture of tailings particles, cement particles, ettringite, ice crystale, C-S-H gel, unfrozen water).

Studies [37,38,39] have shown that under the condition of positive-temperature curing, the strength of CTB comes from the interaction force between the cementation force of hydration products such as C-S-H gel, Aft, and CH on tailings particles. Under the action of continuous load, the micropores and microcracks in CTB are closed, and the aggregate particles and cementation bodies (the hydration products and aggregate particles are cemented) become denser. With the increase in pressure, the stress surpasses the cementation force between the cemented bodies, causes cracks, and then subsequently expands until failure. Under negative-temperature curing conditions, frozen CTB is a four-phase complex composed of aggregate particles, ice, water, and gas. In contrast to the positive-temperature curing condition, ice participation makes its deformation and failure mechanism more complex. The failure process of frozen CTB is briefly investigated and explained in conjunction with Figure 9.

According to the analysis, the failure process of frozen CTB can be separated into four stages, which correspond to (a)–(d) in Figure 9. In the first stage, under the frozen environment, the CTB is a four-phase mixture of solid–liquid–gas and ice, including tailings particles, nonhydrated cement particles, hydration products (mainly AFt and C-S-H gel), solid-phase ice, unfrozen water, and pores (not indicated in Figure 9). At this stage, The CTB skeleton, which is the main source of CTB strength, is formed at this stage by the hydration products, and the cementation ability of the hydration products and the cementation force of the ice work together. In the second stage, there is heterogeneity in the distribution of particles inside CTB, and relatively low-density areas occur locally. Due to the existence of unfrozen water and pores, the compression of the CTB skeleton tends to be compact, and solid particles slip from high-density to low-density areas. When the load exceeds the ultimate strength of the solid-phase ice, the ice body ruptures, the large ice body cracks and slips into small ice bodies, and a portion of the solid-phase ice melts into liquid water under the pressure, which is further dispersed with the assistance of unfrozen water. This is manifested as a high strain in the CTB. In the third stage, with further increase in the load, the pressure induces a small part of the ice body to melt, the solid particles continue to slip to the low-density area, and the CTB skeleton becomes denser. When the load reaches the ultimate strength of the cohesive force of the hydration product gelled, the gelled body fails, manifested by the CTB reaching peak stress and the initiation of microcracks. In the fourth stage, the ice-cementation force and the cohesion of the hydration product colloid are overcome under the action of the load, the solid particles appear reverse slip, tensile stress still needs to overcome the ice cementation force and part of the cohesion of the colloid. The expression is CTB cracks extension, but still has high strength; that is, strain-softening. It is important to note that when the curing time is short, the ultimate strength of the ice’s cementitious body is greater than that of the cement-hydration product’s cementitious body, and the order of occurrence of the second and third stages of the CTB failure process is switched.

Furthermore, compared to normal-temperature curing conditions, the strength development and deformation behavior of CTB under negative-temperature conditions are more complex due to the complexity of pore ice and its dependence and sensitivity to temperature, and the deformation behavior of CTB also changes with curing time, which affects the ice content of CTB. Besides temperature and CTR, there are many other factors affecting CTB strength that are not considered in this paper, such as tailings grain-size gradation, cement type, and additive type. Because of the varied mix components of the filler in different mines, the effect of negative curing temperature on the CTB behavior of diverse mineral admixtures warrants additional investigation.

## 5. Conclusions

The effect of curing temperature on CTB properties was investigated in this study. The mechanical properties and microstructure of CTB with different CTR (1:4, 1:8, 1:12, 1:16, and 1:20) cured at 20 °C, 5 °C, −5 °C, and −20 °C for 3, 7, and 28 days, respectively, were tested. The strength development characteristics and deformation behavior of CTB under positive- and negative-temperature conditions were compared and analyzed, and the following conclusions were obtained.

(1) There is significant variability in the effects of positive- and negative-temperature curing conditions on the properties of CTB. The inhibitory effect of low-temperature on the hydration reaction and the phase transition of pore water is the essential causes of the mechanical and physical properties of CTB.
(2) The strength of CTB decreases when the temperature decreases under positive temperature curing conditions. However, when the curing temperature is subzero, CTB exhibits higher early UCS, and the lower the temperature, the higher the early UCS, which is proportional to the ice content of CTB. The early UCS of CTB is aided by the negative-temperature curing condition, but not by the long-term UCS.
(3) The sub-zero curing temperature forces a phase change from liquid water to pore ice. The deformation characteristics of frozen CTB become more complicated due to the participation of pore ice. After reaching the strain peak, the frozen CTB still retains high strength and good compressibility with strain-hardening or strain-softening phenomena.
(4) With the decrease in the curing temperature, the failure characteristics of CTB evolve from splitting failure to shear failure and then to plastic tensile failure, which is a kind of frozen CTB-specific failure characteristic. Moreover, this study discussed and explained the microscopic failure process of plastic tensile damage of CTB at sub-zero curing temperature by combining XRD and SEM techniques.

## Figures and Tables

**Table 1:** Basic physical properties of experimental tailings.

Proportion	Bulk Density (t/m³)	Compacted Density (t/m³)	Porosity (%)	Natural Angle of Repose (°)	20 °C Permeability Coefficient (cm/h)
2.59	1.20	1.60	57.03	35.37	5.21

**Table 2:** Chemical composition of the OPC.

Composition	SiO_2_	Al_2_O_3_	TFe	SO_3_	MgO	CaO	Others
Content(%)	24.94	5.78	2.17	1.01	2.2	51.27	12.63

**Table 3:** Summary of the mix compositions of the samples prepared.

Test No.	Curing Temperature	Mass Concentration	CTR	Curing Conditions	Curing Time (Days)
A	20 °C	73%	1:4, 1:8, 1:12, 1:16, 1:20	sealed undrained unstressed	3, 7, 28
B	5 °C				3, 7, 28
C	−5 °C				3, 7, 28
D	−20 °C				3, 7, 28

## Data Availability

The data presented in this study are available on request from the corresponding author.
